# Identification and functional characterization of Toll-like receptor 2–1 in geese

**DOI:** 10.1186/s12917-015-0420-y

**Published:** 2015-05-14

**Authors:** Yanhong Yong, Shaofeng Liu, Guohong Hua, Rumin Jia, Yuntao Zhao, Xingmin Sun, Ming Liao, Xianghong Ju

**Affiliations:** Department of Veterinary Medicine, Guangdong Ocean University, Zhanjiang, 524088 China; Department of Animal Science, Guangdong Ocean University, Zhanjiang, 524088 China; Center of Modern Biochemistry, Guangdong Ocean University, Zhanjiang, 524088 China; Department of Infectious Diseases and Global Health, Tufts University Cummings School of Veterinary Medicine, North Grafton, MA 01536 USA; MOA Key Laboratory for Animal Vaccine Development, Key Laboratory of Zoonoses Control and Prevention of Guangdong, College of Veterinary Medicine, South China Agricultural University, Guangzhou, 510642 China

**Keywords:** Goose, TLR2-1, NF-кB, *Mycoplasma*, *Salmonella*, Infection

## Abstract

**Background:**

Toll-like receptor 2 (TLR2), an important pattern recognition receptor, activates proinflammatory pathways in response to various pathogens. It has been reported in humans and chicken, but not in geese, an important waterfowl species in China. Since some vaccines stimulate robust immune responsesl in chicken but not in geeeses we speculated that their immune systems are different.

**Results:**

In this study, we cloned the goose TLR2-1 gene using rapid amplification of cDNA ends (RACE)and showed that geese TLR2-1 encoded a 793-amino-acid protein, containing a signal secretion peptide, an extracellular leucine-rich repeat domain, a transmembrane domain and a Toll/interleukin-1 receptor signaling domain deduced from amino acid sequence. TLR2-1 shared 38.4%–93.5% homology with its homologues in other species. Tissue expression of geese TLR2-1 varied markedly, and was higher in kidney, cloacal bursa, skin and brain compared to other organs/tissues. HEK293 cells transfected with plasmids carrying goose TLR2-1 and NF-κB-luciferase responded significantly to stimulation with *Mycoplasma fermentans* lipopeptide. Furthermore, geese infected with *Mycoplasma gallisepticum* (MG) and *Salmonella enteritidis* (SE) showed significant upregulation of TLR2-1 in both *in vivo* and *in vitro*.

**Conclusion:**

Geese TLR2-1 is a functional homologue of TLR2 present in other species and plays an important role in bacterial recognition in geese*.*

## Background

Toll-like receptors (TLRs) have emerged as a major component of the vertebrate pattern recognition receptor (PRR) repertoire, and can recognize conserved pathogen-associated molecular patterns (PAMPs) of bacteria, fungi, viruses and parasites as well as some self-ligands, such as host DNA in immune complexes and the malarial pigment hemozoin, (hemoglobin digested by malarial parasites), but not commensal microbes [1-3]. Upon activation by an agonist, TLRs induce the expression of a wide range of immunoregulatory and effector molecules [4-6], which take part in the regulation of immune cell maturation and the clearance of pathogenic microorganisms [5,7-9].

In humans and mice,four TLRs (TLR4, TLR2, TLR9 and TLR5) are responsible for recognition of antigens from Salmonella enteritidis (SE),a Gram-negative bacteria, as well as via TLR4, TLR21 and TLR2-1 in Chicken [10,11].Furthermore, TLR2 dimerizes with TLR1 or TLR6, enabling it to recognize microbial triacyl lipoproteins or diacyl lipopeptides found in mycoplasma, lipoteichoic acid of Gram-positive bacteria or zymosan of yeasts [12].However, caution is required in the interpretation of these findings as TLR2 may not recognize some agonists at physiologically relevant concentrations [13]. Recently, human TLR10 has also been shown to dimerize with TLR2 enabling activation by triacyl lipoproteins [14]. Chicken chTLR1La and b, and chTLR2a and b have been cloned and expressed in mammalian cells by several groups and were demonstrated to be surface- exposed [15].

It was thought that some diseases, such as Newcastle disease and H5N1 avian influenza, occurred only in domestic avian species, did not appear or had only very low morbidity in waterfowl [16]. However, since 1997, serotype I avian paramyxoviruses (APMV-1) have caused high morbidity and mortality in geese during outbreaks in many provinces of China. Interestingly, recent studies have not determined any obvious variation in the gene sequences of APMV-1 [17]. TLR2 genes has been identified in mammalian and nonmammalian vertebrates including chicken [18-20]. However, it is not known whether geese also carry a homologue of mammalian TLR2 for recognizing the pathogens. In the present study, we cloned and sequenced the goose TLR2-1, analyzed its expression in several tissues and verified its lipopeptide-mediated activation in HEK-293 T cells. Meanwhile, the expression of TLR2-1 in goose tissues after they were challenged with *Mycoplasma gallisepticum* (MG) or *Salmonella enteritidis* (SE) were also studied.

## Results

### Cloning of goose TLR2-1

**Figure 1:**
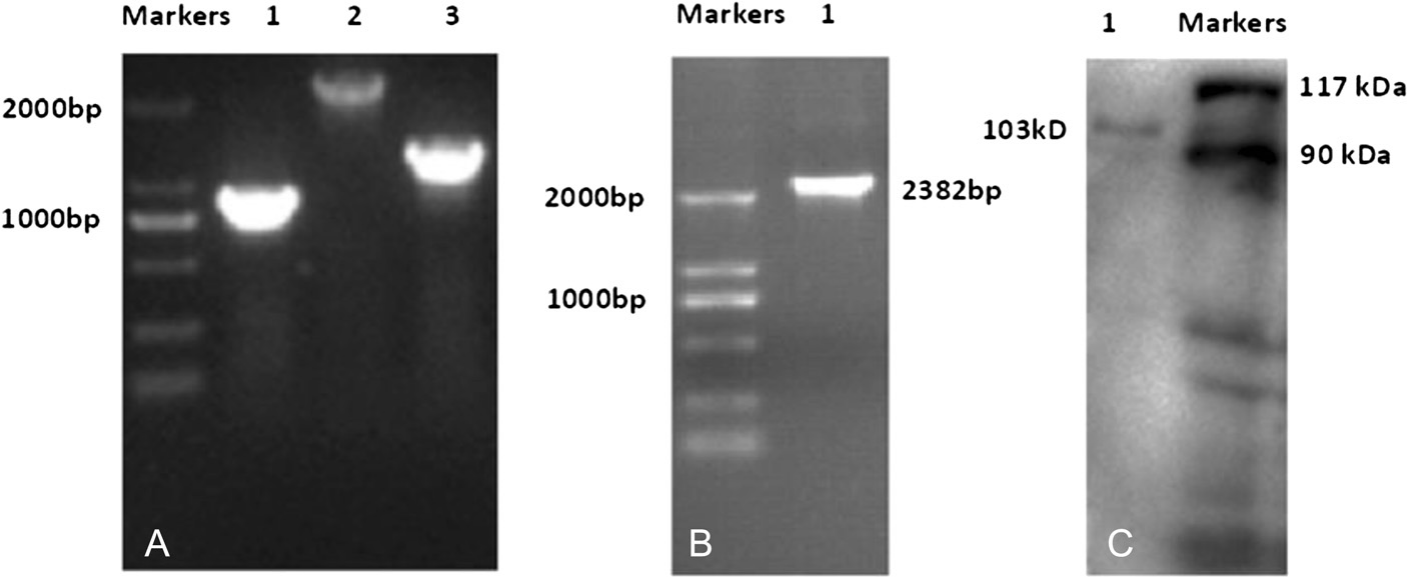
The results of goose TLR2-1 gene cloning and eukaryotic expression. **(A)** Electro-phoresis of RT-PCR and RACE-PCR products of the goose TLR2-1 gene. Lane 1 represents the 5′-RACE-PCR product, a 930-bp fragment. Lane 3 represents the 3′-RACE-PCR product, a 1,201 bp fragment. Lane 2 represents the homologous cDNA fragments from chicken TLR2-1, forming a band of 2150 bp. **(B)** Electrophoresis of the full-length TLR2-1encoding an open read frame, a fragment of 2,382 bp. **(C)** Immunoblotting of gTLR2-1 expressed in HEK293 cells, showing a 103-kDa band.

R fragments of various sizes were subcloned into the vector PMD18-T, and their sequences were determined. One of the clones was found to contain a sequence similar to the TIR domain of chicken TLR2s. A fragment of 1,201 bp was obtained by 3′-RACE-PCR. A 930-bp fragment was then amplified by 5′-RACE-PCR. A 3,119 bp nucleotide sequence representing the full-length, complete cDNA of the goose TLR2-1 gene was obtained from overlapping cDNA fragments. The full-length cDNA harbored an ORF of 2,382 bp, encoding a protein of 793 amino acids in length (Figure 1). We aligned the determined nucleotide sequence from geese with sequences in Genebank by Blast search tool of National Center for Biotechnology Information (NCBI), and named the present gene as gTLR2-1. The sequence was submitted to GenBank and assigned the accession number GenBank: JN982474. The untranslated regions (UTRs) were 359 bp and 378 bp for the 5′-UTR and 3′-UTR, respectively.

**Figure 2:**
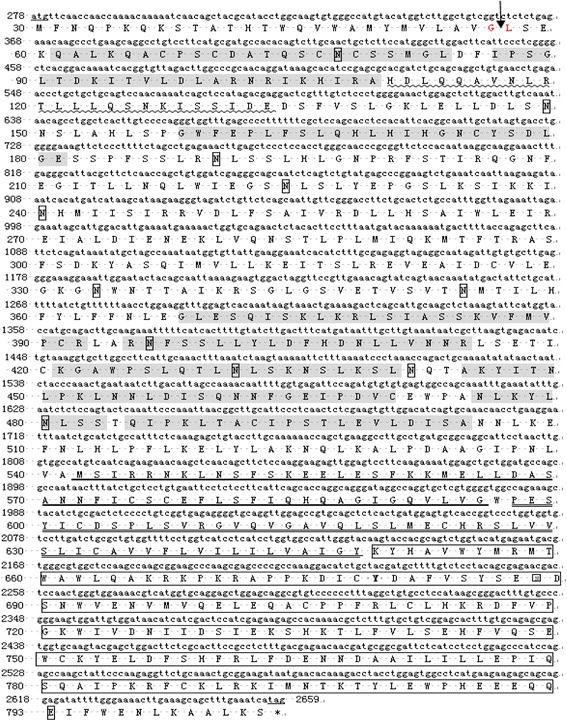
Nucleotide and deduced amino acid sequence of goose TLR2-1. Complete sequence of the full-length gTLR2-1 was deposited in the cDNA library (GenBank: JN982474). Translated amino acid sequence is shown under the nucleotide sequence. Numbers to the left of each row refer to nucleotides. The cleavage site for the putative signal peptide is indicated by an arrow. Leucine-rich repeat (LRR) domains are shaded in grey. Potential N-glycosylation sites are shown as square boxes. The initiation codon (atg) is underlined. The predicted transmembrane segment is double underlined. The translational stop site is indicated by an asterisk. The cysteines critical for the maintenance of the structure of leucine-rich repeat C-terminal (LRR-CT) are marked with coarse underlines. LRR-TYP motif is shown by wave lines. TIR motif is shown by a long box.

**Figure 3:**
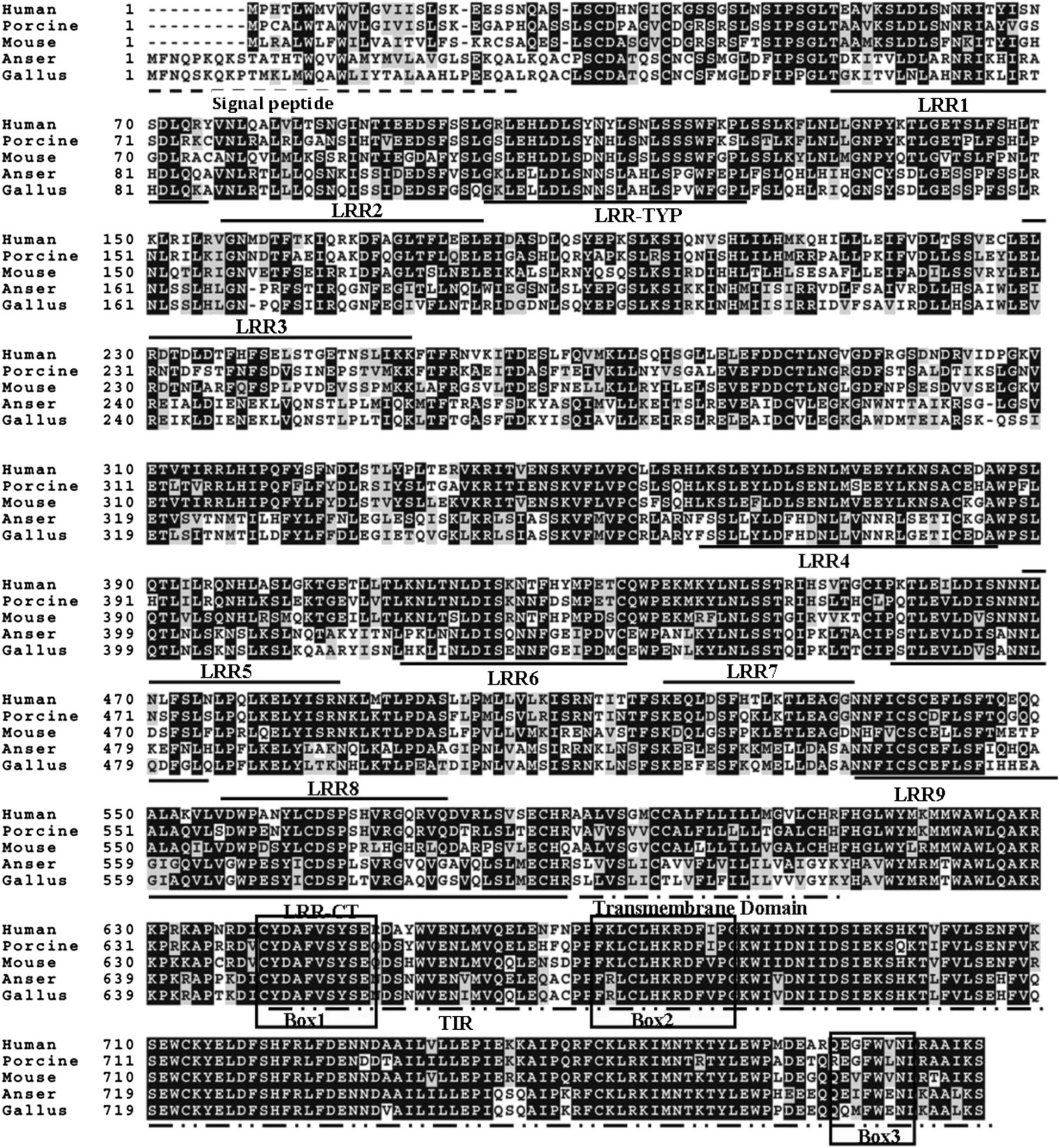
Multiple alignment of TLR2-1 Toll/interleukin 1 receptor (TIR) domains from different species. TIR domains of TLR2 protein sequences were determined by the SMART program and aligned using ClustalW. Three conserved boxes (box 1, 2 and 3) are framed. LRR, leucine-rich repeats; LRR-CT, leucine-rich repeat C-terminal; LRR-TYP, typical LRRs.

Prediction of protein domains revealed that the putative amino acid sequence consisted of a signal peptide sequence encompassing the first 28 amino acid residues of the N-terminal region, nine leucine-rich repeat (LRR) domains, a leucine-rich repeat C-terminal (LRR-CT) domain, a transmembrane domain and a 144-amino-acid Toll-interleukin-receptor (TIR) domain at positions 649–793 of the carboxy-terminus (Figure 2). Highly conserved three subsections of the TIR domain (boxes 1, 2 and 3) is shown in Figure 3.

### Tissue distribution analyzed by quantitative RT-PCR

**Figure 4:**
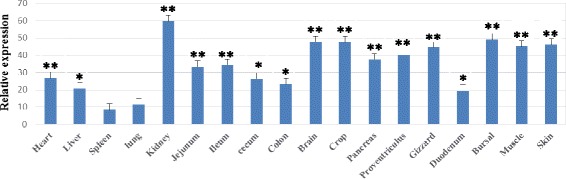
Relative gTLR2-1 expression levels in goose tissues. The expression of the gTLR2-1 gene in 18 different tissues was analyzed by quantitative RT-PCR. The data are presented as mean +/− SD, and were normalized using the β-actin gene and calculated by the delta Ct method. We compared the gTLR2-1 expression in indicated tissues to that in spleen (the tissue with the lowest expression). “*” and “**”indicates that p ≤ 0.05 and p ≤ 0.01 for each test respectively.

The expression of gTLR2-1 in geese organ tissues was analyzed by quantitative RT-PCR. The level of gTLR2-1 mRNA was highest in kidney, and moderate expression levels were seen in bursa, intestine, brain, stomach and heart. The expression levels in the spleen and lung were relatively low (Figure 4).

### MALP-2 stimulation of HEK-293 cells expressing goose TLR2-1 results in NF-κB activation

**Figure 5:**
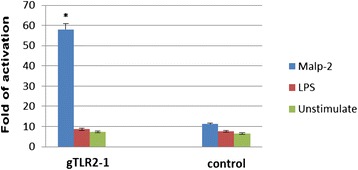
Expression of goose TLR2-1 in HEK293 cells results in NF-κB activation after stimulation of cells with MALP-2 or LPS. Cells were transfected with NF-κB firefly luciferase reporter and normalizing plasmid alone or with constructs expressing goose TLR2-1. After 16 hours, medium was replaced either with fresh medium alone or medium containing MALP-2 (2001) at 50 ng/ml or LPS at 20 ng/ml. NF- κB activation levels were measured after six hours by a dual luciferase reporter assay. The data are presented as mean +/− SD, and statistical analysised by One-Way Anova.

Goose TLR2-1 was cloned downstream from a pCMV promoter with a His-tag fused at the carboxyl terminus. Expression of gTLR2-1 was confirmed by Western blot analysis of HEK-293 cells transiently transfected with the gTLR2-1 expression plasmid, showing a protein band with an expected size of 103 KD (Figure 1). As the activation of TLRs results in its interaction with adaptor molecules and downstream signaling, leading to upregulation of genes with NF-κB elements, a NF-κB luciferase reporter system was used to determine whether the expressed recombinant goose TLR could be activated by exogenous macrophage-activating lipopeptide (MALP-2, 10 ng/ml) or Lipopolysaccharides (LPS, 50 ng/ml). As shown in Figure 5, the activation of NF-κB increased over 58-fold in HEK-293 T cells that were transiently transfected with plasmid encoding gTLR2-1 and stimulated with MALP-2 for 6 hours., However, this phenomenon was not seen with LPS stimulation. In addition, transfection with gTLR2-1–encoding plasmid resulted in a detectable amount activation of NF-κB (compared with cells transfected with reporter/normalizing plasmids alone) in the absence of MALP-2 stimulation, suggesting that ectopic expression of gTLR2 in this human cell line, can mediate a basal level of activation.

### MG and SE infection induced the upregulation of TLR2-1 *in vivo*

**Figure 6:**
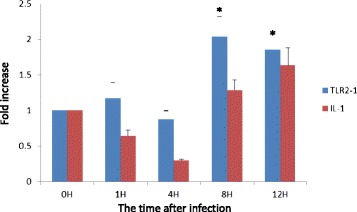
Expression of goose TLR2-1 and IL-1β in PBMCs after stimulation by MALP-2. Goose PBMCs were isolated and cultured with RPMI 1640. After cells were stimulated for 30 min by 50 ng/ml of MALP-2, the expression of TLR2-1 or IL-1β were measured at 0, 1, 4, 8 and 12 h after the end of the exposure. The data are presented as the mean ± SD, and statistically analyzed by one-way ANOVA. “*” indicates that p ≤ 0.05 for each test.

Exposure to Malp-2 significantly increased the expression of TLR2-1 in PBMCs at 8 h and 12 h post-exposure (p < 0.05), with the peak value attained at 8 h after the end of stimulation (Figure 6), while IL-1 transcription showed a much lee significant increase when compared with TLR2-1.

**Figure 7:**
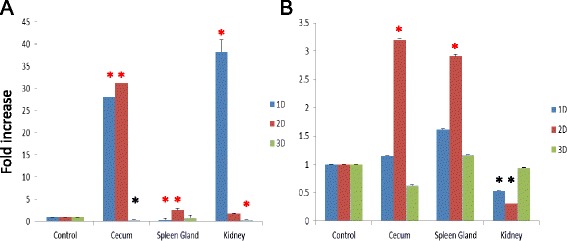
Expression of goose TLR2-1 in indicated tissues after infection with *Mycoplasma gallisepticum* **(A)** *Salmonella enteritidis* **(B)** and phosphate buffered saline (PBS) as a mock. 4-week-old geese were infected with 0.2 ml Mycoplasma gallisepticum (2 x 107 CFU/ml) and 0.5 ml *Salmonella enteritidis* (1 x 106 CFU/ml) with intrapleural or intramuscular injections, respectively. The expression of TLR2-1 mRNA in the cecum, spleen gland and kidneys of infected geese were detected by qRT-PCR. The data are presented as the mean ± SD, and statistically analyzed by one-way ANOVA. “*” and “*” indicate that p ≤ 0.05 and p ≤ 0.01 for each test, respectively.

MG infection significantly upregulated the expression of TLR2-1 in the cecum at days 1 and 2, in the spleen gland on day 2 and in the kidneys on day 1 (Figure 7-A). Furthermore, SE infection increased the expression of TLR2-1 in the cecum and spleen gland on day 2 (Figure 7-A), with approximately 3-fold increase in both tissues (p < 0.05). Interestingly, a downregulation of TLR2-1 was also observed in the kidney on both days 1(0.51-fold compared to control geese) and 2 (0.30-fold compared to control geese).

## Discussion

The role of TLRs in the immune response to pathogenic bacteria and viruses have been studied intensively possibly due to their ability to recognize pathogens and initiate development of an immune response. Currently, 13 TLRs in humans and 10 TLRs in chicken have been described but less is known about TLRs in geese. In the present study, we for the first time cloned and sequenced TLR2-1 cDNA in goose, and found that the full-length TLR2-1 cDNA (3,119 bp) contained a 5′ untranslated region (359 bp), an open reading frame (2,382 bp) encoding 793 amino acids and a 3′ untranslated region (378 bp). The sequence homologies of nucleotides and amino acids were 39.6%, 51.9%, 83.6 and 93.5% respectively in comparison with human, pig, turkey and duck, and the amino acid sequence was 84.6% homologous with chicken TLR2-1 (Table 1 and Table 2). We also found that TLR2-1 played an role in the recognition of *Mycoplasma fermentans* lipopeptide, MG, SE, and induced the activation of NF-κB.

**Table 1:** **Comparison of gTLR2-1 amino acid sequence homology with chicken’s TLRs** ^**a**^

**Species**	**gTLR2-1**	**ChTLR1LA**	**ChTLR1LB**	**ChTLR2A**	**ChTLR2B**	**ChTLR3**	**ChTLR4**	**ChTLR5**	**ChTLR7**	**ChTLR15**	**ChTLR16**	**ChTLR21**
gTLR2-1	100%											
ChTLR1LA	29.4%	100%										
ChTLR1LB	28.3%	71.6%	100%									
ChTLR2A	84.6%	28.9%	29.1%	100%								
ChTLR2B	72.7%	27.1%	26.6%	81.7%	100%							
ChTLR3	20.3%	21.5%	18.6%	20.4%	20.6%	100%						
ChTLR4	21.5%	18.1%	17.6%	21.2%	20.9%	19.4%	100%					
ChTLR5	21.3%	21.2%	19.4%	20.4%	20.3%	21.7%	20.3%	100%				
ChTLR7	22.3%	21.3%	20.6%	22.3%	21.2%	22.3%	22.4%	21.9%	100%			
ChTLR15	24.7%	26.2%	23.8%	24.1%	24.1%	16.1%	19.5%	18.0%	20.0%	100%		
ChTLR16	28.6%	97.4%	73.9%	29.0%	27.4%	20.6%	19.5%	20.6%	20.9%	24.2%	100%	
ChTLR21	24.3%	23.6%	20.7%	23.9%	24.8%	20.4%	19.5%	21.0%	20.2%	20.4%	22.3%	100%

^a^Homology of goose TLR2-1 with TLR2 of various species at the nucleotide level. Goose (G) TLR2-1 nucleotide sequence were compared with those of chicken (Ch) TLR1LA (GenBank: JF823976.1), TLR1LB (GenBank: FJ915342.1), TLR2A (GenBank: FJ915424.1), TLR2B (GenBank: FJ915378.1), TLR3 (GenBank: JF273967.1), TLR4 (GenBank: JQ711152.1), TLR5 (GenBank: JX573117.1), TLR7 (GenBank: FJ915590.1), TLR15 (GenBank: FJ915250.1), TLR16 (GenBank: EF413646.1) and TLR21 (GenBank: NM_001030558.1).

**Table 2:** **Comparison of TLR2-1 amino acid sequence homology between various species** ^**a**^

**Species**	**Goose**	**Chicken**	**Turkey**	**Bird**	**Duck**	**Human**	**Mouse**	**Pig**	**Cattle**	**Fish**
Goose										
Chicken	84.6%									
Turkey	83.6%	91.7%								
Bird	79.4%	78.3%	77.4%							
Duck	93.5	82.3%	81.4%	77.7%						
Human	52.9%	52.7%	52.9%	52.3%	52.7%					
Mouse	50.2%	49.7%	50.3%	50.6%	49.7%	71.2%				
Pig	52.5%	51.4%	51.4%	51.4%	51.9%	78.0%	68.6%			
Cattle	51.6%	51.2%	51.4%	51.0%	51.2%	78.0%	67.3%	80.9%		
Fish	38.4%	37.5%	37.6%	38.2%	38.4%	38.6%	39.2%	39.1%	40.4%	

^a^ Homology of goose TLR2-1 with TLR2 of various species at the amino acid level. Goose TLR2-1 mRNA and encoded amino acid were compared with those of chicken (GenBank: NP_989609.1), turkey (GenBank: ACS92625.1), zebra finch (GenBank: XP_002198506.2), duck (GenBank: ACS92627.1), human (GenBank: NM 003268), pig (GenBank: BAC99316), cattle (GenBank: AAT48487), mouse (GenBank: AF186107) and *Takifugu rubripes* (GenBank: AAW69370).

The organization of LRRs provides the protein structures for specific interaction with the respective PAMP and varies between TLR groups and species. The ectodomains of TLRs comprise 19–25 tandem copies of LRRs, which are thought to be involved in the recognition of various microbial ligands [21]. In contrast, the cytoplasmic portion of TLRs, the TIR domain, which has homology with the cytoplasmic domain of human IL-1, is the most conserved between the TLRs and has a very ancient evolutionary origin [22]. The TIR domain is also involved in bimolecular interactions with MyD88, an adaptor protein necessary for signal transduction [23]. Based on the predicted amino acid sequence, gTLR2-1 consists of an extracellular scaffold of LRR domains and the intracellular TIR domain. Variability in amino acid sequences is present in the N-terminal region, resulting in 12 LRR domains, as in the chicken. Sequence analysis in goose indicated high conservation of the three subsections of the TIR domain (boxes 1, 2, 3) known to be important in signaling (boxes 1 and 2) and receptor localization (box 3) [24,25]. Overall, the gTLR2-1 proteins share 85.1% identity with chicken.

TLR2 mRNA expression has been reported to be relatively moderate in circulating leukocytes and the spleen, moderate in the ovary, lung, pancreas and placenta, and low in brain, colon, heart, kidney, liver, prostate, small intestine, testis, skeletal muscle and thymus in humans [26]. The pattern of gTLR2-1 mRNA expression was similar to that in humans according our study. It is notable that gTLR2-1 mRNA was higher in skin, intestine and immune tissue, which are all exposed to the external environment. We hypothesize that this characteristic expression pattern of gTLR2-1 is associated with the habitat of the goose, and that frequent contact with microorganisms in the water may increase the expression of TLRs due to frequent challenge by pathogenic bacteria. However, further study is needed to analyze the expression characteristics of TLRs in skin exposed to water containing different bacterial loads.

In addition to TLR2, TLR4 expression has also been reported in a wide range of tissues and is responsible for recognition of PAMPs including LPS of Gram-negative bacteria [27]. Each of these receptors plays an important role in defense against microbial infection. TLR2-deficient macrophages have been shown to be hyporesponsive to *Staphylococcus aureus* peptidoglycan, and C3H/Hej mice with missense mutations of the TLR4 gene were highly susceptible to Gram-negative bacterial infection [28-30]. Therefore, it has been suggested that cooperation between TLR2 and TLR4 is important in the detection of microorganisms and in inducing inflammatory responses. However, canine TLR2 mRNA was detected in skin in the absence of TLR4 mRNA expression [27], and similar findings were reported in human keratinocytes [31]. In our study, goose TLR2-1 mRNA expression was higher in skin and muscle, which form a first line defense against a variety of pathogens, especially Gram-positive bacteria. In future study, we will explore the expression profile of TLR4 and its interaction with TLR2 in the skin of the goose.

TLR2 acts as a pattern recognition receptor and signals the presence of various bacteria through the activation of NF-κB, not only in human and mouse, but also in chicken [32-35]. Since the TIR domain of gTLR2-1 was highly homologous to that of human, as well as to oviparous vertebrates, and the amino acid residues responsible for signaling were also apparently conserved, we presumed that goose TLR2-1 may be analogues of mammalian TLR2. We therefore tested whether mycoplasma components can activate NF-κB via the TLR pathway. LPS and MALP-2, the traditional representative ligands of TLR4 and TLR2 in mammals [36-40], were used in the present study. We found that MALP-2 but not *E. coli* LPS served as a ligand for NF-κB activation in HEK293 cells expressing gTLR2-1, indicating that gTLR2-1 was an analogue of mammalian TLR2 and plays an role in the recognition of MALP-2.

Mycoplasmas were first isolated from geese by Kosova and Djurisic [41], and further studies also isolated Mycoplasmas successfully from geese in association with various conditions such as airsacculitis and peritonitis [42-44]. Intratracheal infection of goslings or goose embryos with Mycoplasma and Acholeplasma strains resulted in airsacculitis [45]. Other studies have shown that *Mycoplasma cloacae* and *Mycoplasma anseris* induced a fall in egg production and increased embryo mortality [46]. The present results found that not only MG, but SE infections, induced the expression of TLR2-1 in goose in a tissue- and time-dependent manner, which suggested that the immune system of the goose may recognize the PAMP of MG and SE via TLR2-1. But the fold changes in SE challenge geese was smaller than the case of MG,may due to the PAMPS of SE generally accepted to include LPS, flagellin, CpG-ODN, which may not be the major ligands of TLR2 in mammals and chickens.

As mentioned above, the dimerization of TLRs is important in the recognition of pathogens. Synthetic MALP-2 induces the activation of NF-κB in HEK-293 cells that are co-transfected with chTLR2a and b [47], and its exogenous agonist does not induce the activation of NF-κB when transfected cells express chTLR2a, b, or chTLR1La or b separately [35]. However, the reverse was observed when expression of chTLR2b/chTLR1La was combined, and in this case activation was induced both by synthetic MALP-2 and PAM3. Thus, although there are anomalies between these studies, they do demonstrate that chTLR1Ls interact with chTLR2s and can recognize agonists identical to those of the mammalian dimers of TLR2 with TLRs1, 6 and 10. However in geese, further study is needed to explore the interaction between TLRs and the signal pathways are activated by pathogen invasion in particular.

## Conclusions

We cloned goose TLR2-1, examined its expression pattern, investigated the gTLR2-1–mediated signal pathway and the expression of gTLR2-1 after pathogenic infection. These results suggest that gTLR2-1 plays a role in the recognition of pathogen-associated molecular patterns in bacteria, and in particular *Mycoplasma* spp. infections.

## Methods

### Animal

Magang geese were purchased from Qingyuan Livestock Co. Ltd. (Guangdong, China). For cloning TLR2-1 and harvesting peripheral blood mononuclear cells (PBMCs), 30-week-old geese were used. For artificial infection experiments, 1-day-old geese were obtained and allowed to acclimatize for approximately 3 weeks. All geese were housed in isolators. Geese were found serologically negative for *Mycoplasma gallisepticum* and *Salmonella enteritidis* by rapid serum agglutination test and enzyme-linked immunosorbent assay, respectively. Geese were given access to water *ad libitum*. Diet was formulated according to the recommended nutrient allowances for this breed of goose.

### Statement of ethical approval

All animal experiments were conducted under the guidance of the CDC’s Institutional Animal Care and Use Committee in an Association for the Assessment and Accreditation of Laboratory Animal Care International accredited facility. The protocol was approved by the Committee on the Ethics of Animal Experiments of the Guangdong Ocean University (Permit Number: 201–1225). All surgery was performed under sodium pentobarbital anesthesia, and every effort was made to minimize suffering.

### Cells culture

Human embryonic kidney (HEK) 293 cells were purchased from Type Culture Collection of the Chinese Academy of Sciences, Shanghai, China. Cells were maintained in Dulbecco’s modified Eagle’s medium (DMEM) supplemented with 10% fetal bovine serum. Goose PBMCs were isolated by density gradient centrifugation using LTS-1077 according to the manufacturer’s protocol (density = 1.077 g/mL; TBD, Tianjin Haoyang Biological Manufacture Co., Ltd., Tianjin, China). In brief, 2 mL of whole blood was diluted with 2 mL Hank’s medium and layered over LTS-1077, before centrifugation at 225 × g for 15 min at room temperature. PBMCs were collected from the layer between the 1077 layer and the serum, washed twice in Hank’s medium, resuspended and counted. Then, PBMCs were resuspended in culture medium (RPMI 1640, GIBCO) at a concentration of 2 × 10^5^ cells/mL, and 0.5 mL of cell suspension was seeded in each well of 24-well tissue culture plates and incubated at 37°C in a carbon dioxide incubator containing 95% air and 5% CO_2_ at a humidity of 100%.

### Cloning of TLR2 homologue in geese (gTLR2-1)

Total RNA was extracted from the spleens of three Magang geese using RNAiso Plus (TaKaRa Biotechnology Co. Ltd., Dalian, China) according to the manufacturer’s instructions. To avoid contamination with genomic DNA, total RNA samples were treated with RNase-free DNase l (TaKaRa). To clone the goose TLR2-1 (gTLR2-1) gene, PCR primers (Table 3) were designed based on the well-conserved nucleotide sequences of TLR2-1 from chicken (*Gallus gallus*; GenBank: NP_989609.1), duck (*Anas platyrhynchos*; GenBank: FJ477862) and turkey (*Meleagris gallopavo*; GenBank: FJ477860). The PCR conditions consisted of an initial denaturation at 94°C for 5 min; 35 cycles of denaturation (94°C for 30 s), annealing (57°C for 30 s) and extension (72°C for 1 min) at, and; and a final elongation step at 72°C for 7 min. The PCR products were ligated into the pMD18-T vector (Takara, China) and sequenced by Shanghai Invitrogen Biotechnology Co., Ltd.

**Table 3:** **Primer Pairs designed for TLR2-1 gene**

**Primer name**	**Primer sequence(5′-3′)**	**Annealing temp (°C)**	**Product size**
gTLR2-1 F	GCCACTCAGTCATGCAACTGCT	58	2100 bp
gTLR2-1R	CTATGACTTCAAGGCTGCTTTCAAG		
gTLR 2-1qF	ATGTGTGTGAGTGGCCAGCA	62	200 bp
gTLR 2-1qR	TTGAGAAATGGCAGATGCAG		
β-actin F	CGGCAACGAGCGGTTCAGGT	61	150 bp
β-actin R	GGGTACATGGTGGTGCCGCC		
gIL-1βF	CCTGCCTCTGTCTTCAGAAGAAGCCTCGTC	62	257 bp
gIL-1βR	CCTCCTCCAGGAGAGCGCTCAGGTCGCT		
GTLR2-1 F	GACCATGGTCAACCAACCAAAAC	58	2382 bp
GTLR2-1R	TCATGATTTCAAAGCTGCTTTCAAG		
3′RACE-PCR	TTCAGCACCAGGCAGGGATAGGC	68	1201 bp
5′RACE-PCR	GCCGTGAATGTGGAGGTGCTGGA	70	930 bp

*Abbreviations*: F, forward; R, reverse; RACE, rapid amplification of cDNA ends; UPM, Universal Primer A Mix; qF, forward primer for qRT-PCR; qR, reverse primer for qRT-PCR.

In order to get the full-length cDNA of TLR2-1 from geese, the 5′- and 3′-SMART rapid amplification of cDNA ends (RACE) PCR (Clontech, Clontech Laboratories, Inc., Mountain View, CA) was performed according to the manufacturer’s instructions. Gene-specific primers used for the amplification of RACE cDNA fragments (Table 3) were designed based on the above partially sequenced gTLR2-1. Gene amplifications were performed by touchdown PCR with gene-specific primers to obtain specific PCR products. PCR amplicons were then cloned into a plasmid vector for nucleotide sequencing as described above.

### Gene analysis

The determined DNA sequences were assembled and edited with DNAman software (Version 3.0, Lynnon, BioSoft). The sequences of the predicted open reading frames (ORFs) or the TIR domains were compared with other sequences by the BLAST program (http://www.ncbi.nlm.nih.gov/BLAST/). TLR family members from *Homo sapiens*, *Sus scrofa*, *Mus musculus*, *Gallus gallus*, *Bos gaurus* and *Takifugu rubripes* were identified in the database (http://www.ncbi.nlm.nih.gov/Genbank/). Alignment of the amino acid sequences and unrooted phylogenic analysis of TLRs were performed using the ClustalW program (http://www.ddbj.nig.ac.jp/search/clustalw-j.html). Protein N-terminal signal peptide structures were predicted by SignalP 3.0, and N-glycosylation sites were predicted by the NetNGlyc1.0 Server. Transmembrane structures were predicted by Server v. 2.0. Conserved domains were predicted by the SMART service.

### Tissue expression of goose TLR2-1 by qRT-PCR

Total RNA was extracted from geese tissues (from six 10-week-old Magang geese) using RNAiso Plus (TaKaRa) and reverse-transcribed using QUANTITECT® (Qiagen, Germany). qPCR was performed using a SYBR® green polymerase mix (TaKaRa). The primers (Table 3) used for qRT-PCR were designed by using the primer3 software (http://bioinfo.ut.ee/primer3/). To validate qRT-PCR purified products were cloned into pMD19-T (Takara) and sequenced to verify the correct target amplification. PCR products were amplified using a LightCycler 480 (95°C, 15 s; 61°C, 15 s; 72°C, 15 s). The data are presented as mean +/− SD, and were normalized using the β-actin as a house-keeping gene and calculated by the delta Ct method. We compared the gTLR2-1 expression in the indicated tissues with that in the lowest expression tissue. Tissues used for the study included heart, liver, spleen, lung, kidney, jejunum, ileum, cecum, colon, brain, crop, pancreas, proventriculus, gizzard, duodenum, bursa, breast muscle and skin.

### Expression of gTLR2-1

ggTLR2-1 gene sequence encoding the full-length ORF were ligated into the mammalian expression vector, pcDNA3.1/V5-His-TOPO TA (Invitrogen, Shanghai, China). The resulting plasmid was introduced into HEK-293 cells by transfection using Lipofectamine®-2000 (Invitrogen). After 24 hours of transfection, cells were lysed with lysis buffer (1% Nonidet P-40, 10 mM EDTA, 1 mg/ml iodoacetamide, 1 mM phenylmethylsulfonyl fluoride, 50 mM Tris maleate, pH 8.6) at a density of 1 × 10^7^ cells/ml. Proteins of interest were separated by SDS-polyacrylamide gel electrophoresis [48], transferred to nitrocellulose membranes, and subjected to immunoblotting analysis with anti-His antibody (Abcam, Cambridge, UK). The antigen-antibody complex was detected using an ECL Kit (Amersham Pharmacia Biotech, Amersham, UK), following the manufacturer’s protocol.

### Luciferase reporter gene assay

HEK-293 cells were seeded into 96-well plates at 4 × 10^4^ per well in DMEM. The following day, cells were transfected with plasmids as described above using LIPOFECTAMINE®-2000 (Invitrogen). Briefly, cells in each well were transfected with the following plasmids alone or in combination: 60 ng pNF-κB (which expresses firefly luciferase under the control of NF-κB), 0.2 ng PRL-YK (which expresses Renilla luciferase constitutively via a β-actin promoter and serves to normalize transfection variables), 30 ng of plasmids encoding goose TLR2-1-His, and pcDNA3.1 to bring the total of DNA to 120 ng/well. Twenty-four hours post-transfection, cells were stimulated with ligands for 6 hours unless otherwise stated. The cells were washed once with PBS and lysed in passive lysis buffer (Promega, Shanghai, China). Luciferase activity was measured using the DUAL-LUCIFERASE® Reporter Assay System (Promega). Firefly luciferase values were normalized by dividing light output for firefly luciferase by light output for Renilla luciferase. The experiments were repeated three times.

### Expression of TLR2-1 and IL-1 by PBMCs in response to exposure to macrophage-activating lipopeptide (MALP-2)

After the PBMCs had adhered, the culture medium replaced with RPMI 1640 containing 10 ng/ml MALP-2 (a synthetic dipalmitoylated lipopeptide, purchased from IMGENEX, San Diego, CA), incubated for 30 min and then replaced again with RPMI 1640 in the absence of MALP-2. The other wells were mocks that underwent the medium change process without any change in medium content. The RNA of PBMCs after exposure to MALP for 0, 1, 4, 8 and 12 h- was extracted and the TLR2-1 mRNA were detected by qRT-PCR. In order to determine whether MALP-2 exposure could induce an inflammatory process in cultured PBMC of geese, interleukin-1 mRNA levels were also determined in present study. The experiments were repeated three times.

### Animal experiments

To investigate the expression of TLR2-1 in geese challenged by bacteria groups of geese (n = 9) were infected with the *Mycoplasma gallisepticum* (MG) GB44T strain or *Salmonella enteritidis* (SE) 50041 strain (purchased from the China Institute of Veterinary Drug Control) or PBS as controls. The LD50 and infection dose of each pathogen were calculated according to the methods described by Reed & Muench [49]. 27 geese (aged 4 weeks) were divided into three groups randomly (n = 9), and inoculated with 0.2 ml MG (2 × 10^7^ CFU/ml) with an intrapleural injection (MG group) or 0.5 ml SE (1 × 10^6^ CFU/ml) with intramuscular injection (SE group), or PBS at a volume of 0.2 ml (Control group). At 1, 2 and 3 DPI, three individuals from each group were sacrificed and tissues were harvested immediately for RNA extraction (cecum, spleen gland and kidneys lungs).

### Statistical analysis

The relative expression ratios of the target gene in the tested group versus those in the control group were calculated by the 2^-△△Ct^ method using the goose housekeeping gene beta-actin (β-actin; GenBank accession number:M26111) as the endogenous reference gene to normalize the level of target gene expression [50]. Standard deviations were calculated using the relative expression ratios of three replicates for each gene measured. Statistical analyses were performed using SAS version 8 (SAS Institute, Cary, NC). The value of P < 0.05 was significant.

## Abbreviations

TLRs: Toll-like receptors
LRR: Leucine-rich repeats
TIR: Toll-like receptor/ interleukin- 1 receptor
cDNA: complementary DNA
PAMPs: Pathogen associated molecular patterns
PRR: Pattern recognition receptor
LPS: Lipopeptides
MyD88: Myeloid differentiation primary response protein 88
TRIF: TIR-domain containing adaptor protein inducing IFN-β
3′-UTR: 3′-untranslated region
5′-UTR: 5′-untranslated region
ORF: Open reading frame
RACE: Rapid-amplification of cDNA end cDNA
NF-κB: Nuclear factor-kappa B
PCR: Polymerase chain reaction
Amp: Ampicillin
